# Can you read my pokerface? A study on sex differences in dentophobia

**DOI:** 10.1111/eos.12079

**Published:** 2013-09-12

**Authors:** Verena Leutgeb, Sonja Übel, Anne Schienle

**Affiliations:** Department of Clinical Psychology, University of GrazGraz, Austria

**Keywords:** dentophobia, disgust, EMG, heart rate, levator labii

## Abstract

Men and women with dentophobia differ in specific fear contents and underlying brain activity during symptom provocation. Possible sex differences concerning other basic emotions, such as disgust, have undergone minimal investigation. Therefore, we recorded the facial electromyogram from the *musculus levator labii* (as a specific disgust indicator) and the heart rate of 36 individuals with dentophobia (18 women and 18 men with comparable disorder severity) and of 36 non-phobic controls (18 women and 18 men). The participants were asked to look at pictures showing dental treatment scenes, generally fear- and disgust-inducing, as well as pictures with neutral contents. Subsequently they performed an affective picture rating. Independently of sex, phobic subjects relative to controls showed heart-rate acceleration when watching pictures of dental treatment scenes, reflecting a fear reaction. Male and female phobic subjects did not differ in their verbal reports of fear and disgust experienced. However, phobic women showed enhanced disgust-related facial electromyographic activity to dental treatment scenes relative to men. This sex-specific response pattern points to the greater relevance of disgust for the female symptomatology of dentophobia, or it might also be possible that male patients more successfully inhibit disgust reactions during confrontation.

Dentophobia is very common in western societies. The extreme dental fear of such patients leads to avoidance of treatment and deterioration of oral health, which, in turn, reinforces fear (1). In contrast to other types of specific phobia (especially of the animal subtype), dentophobia affects both sexes. Prevalence rates of 4.6% and 2.7% are reported in women and in men, respectively (2). Therefore, dentophobia seems to be suitable for the investigation of sex-specific symptoms.

Most patients report ‘fear of pain’ to be the major concern leading to avoidance of dental treatment (3). However, it has also been discussed that both sexes differ in their concerns (4), with women perceiving a lowered internal control during dental treatment (5).

In recent years, numerous investigations on the psychophysiology of dentophobia, using confrontation with disorder-specific pictures, videos, as well as sounds, have been published (6–14). The majority of these investigations (6–8, 14) reported that dentophobic subjects display heart-rate acceleration when presented with dental treatment scenes, reflecting defensive fear mobilization (15).

It has repeatedly been argued that besides fear, disgust plays a central role in the etiology of psychiatric disorders [for a recent review, see (16)]. Especially, patients with specific phobias of the animal type (spiders) display heightened overall disgust-proneness [e.g. they are more disgusted by poor hygiene and unusual food relative to individuals with no such phobias; (17–19)]. Greater general disgust reactivity has also been reported to be central for patients with blood-injection-injury (BII) phobia (20).

Although dentophobia is classified within the subtype of BII phobia, existing findings on the role of disgust are inconsistent and rather point to a disorder-specific increase of disgust proneness (8). Moreover, in passive picture viewing, dentophobic subjects (both men and women) rated dental-treatment scenes as more disgust-eliciting than did healthy controls, whereas they gave disgust ratings comparable with those of healthy controls for other picture types [e.g. overall disgust-eliciting pictures; (12, 13)].

The prototypical facial disgust expression consists of a nose wrinkle and a retraction of the upper lip, reflecting activation of the *musculus levator labii* (21. 22). There are no published investigations on activation of this muscle region during symptom elicitation in dentophobia. The only published study on dentophobia employing the facial electromyogram (EMG) showed heightened levels of forehead muscle tension during presentation of film clips showing dental drilling or injections compared with neutral scenes (10). However, the study did not employ a non-fearful control group. In previous studies the *musculus levator labii* has proved to be a valid indicator for the disgust reaction in spider-phobic girls (17,23). Therefore, the *musculus levator labii* might also be valuable for studying dentophobia.

The main objective of the current investigation was to compare changes in heart rate and electromyographic activity of the *musculus levator labii* between dentophobic and non-dentophobic men and women and to identify sex-specific psychophysiological reactions. We expected to observe heart-rate acceleration in dentophobic subjects of both sexes during confrontation with dental-treatment scenes, relative to controls. Moreover, we hypothesized higher facial EMG reactivity in women than in men, especially in dentophobic women in response to disorder-relevant stimuli. We also expected to observe higher facial EMG activity in women than in men in response to overall disgust-eliciting scenes and an underlying higher overall disgust-proneness.

## Material and methods

### Participants

Thirty-six right-handed and non-medicated patients (18 women and 18 men) with dentophobia [Diagnostic and Statistical Manual of Mental Disorders, DSM-IV-TR: 300.29; (24)] and 36 non-phobic controls (18 women and 18 men) participated in this study. They were recruited via articles in local newspapers and announcements at the campus, to which 156 persons responded. All were contacted by telephone, and a short interview on the diagnostic criteria of dentophobia and the most common mental disorders was conducted. Subsequently, 86 subjects were invited to a diagnostic session, following which 11 were excluded (see below for criteria). Diagnoses were made by a board-certified clinical psychologist. The patient group did not differ from the control group with respect to age (phobics: mean age ± SD = 29.8 ± 7.2 yr; controls: mean age ± SD = 27.0 ± 6.7 yr). All participants gave written informed consent after the nature of the study had been explained to them. The study was approved by the local Ethics Committee.

### Procedure

First of all, participants underwent a diagnostic session consisting of a clinical interview (Mini-DIPS; 25). Then, a self-constructed interview on diagnostic criteria according to the DSM-IV-TR (24) was conducted. Patients were required to fulfill the diagnostic criteria of dentophobia (including a massive fear reaction, avoidance of dental treatment and restrictions in daily routine/suffering). Patients who had any mental disorder other than dentophobia were excluded. Control-group participants who had any mental disorder were also excluded. All participants filled out the Dental Anxiety Scale (DAS; 26), which consists of four questions targeting subjective anxiety during anticipation and dental treatment (e.g. ‘If you had to go to the dentist tomorrow, how would you feel about it?’). The first question is answered on a five-point scale from ‘I would look forward to it as a reasonably enjoyable experience’ to ‘I would be very frightened of what the dentist might do’. The three remaining questions concern feelings in anticipation of, or during actual treatment (e.g. ‘When you are waiting in the dentist's office for your turn in the chair, how do you feel?’). They are answered on a five-point scale from 1 = ‘Relaxed’ to 5 = ‘So anxious that I sometimes break out in a sweat or almost feel physically sick’. The resulting sum scores range from 4 to 20 and were 9.33 ± 3.17 (mean ± SD) for controls and 17.18 ± 1.8 for phobics (27). According to the authors (26) the DAS shows sufficient reliability (Kuder–Richardson formula coefficient = 0.086) and test–retest stability (correlation coefficient *r* = 0.82). Furthermore, participants filled out the Questionnaire for the Assessment of Disgust Sensitivity (QADS; (28)). This questionnaire consists of 37 items and is a measure of overall disgust-proneness (e.g. ‘You are just about to drink a glass of milk as you notice that it is spoiled’). Items are answered on a five-point scale from 0 = ‘not disgusting’ to 4 = ‘very disgusting’. The questionnaire consists of five subscales: ‘death’, ‘body secretions’, ‘hygiene’, ‘spoilage’, and ‘oral rejection’. Mean scores are calculated for the main scale as well as for the subscales. In healthy samples (28) the mean ± SD score of the main scale is reported to be 2.11 ± 0.57. The internal consistencies of the subscales range from 0.69 to 0.85 (Cronbach's α). The Cronbach's α of the total scale is 0.90 (28). In addition, participants completed the trait scale of the State-Trait Anxiety Inventory (STAI; (29)). This questionnaire is widely used to measure trait anxiety in adults (e.g. ‘I worry too much over something that really doesn't matter’). The scale consists of 20 items, which are judged on 4-point scales (1 = ‘hardly ever’ to 4 = ‘nearly ever’). Sum scores range from 20 to 80. In samples with specific phobia or other anxiety disorders (29) mean ± SD scores are reported to be 53.3 ± 11.4. According to the authors the Cronbach's α is sufficient, with a value of 0.90 (29). Finally, participants filled out the Beck Depression Inventory (BDI; (30)). The BDI is the most employed self-description questionnaire measuring depression symptoms. It consists of 21 items, which are answered according to symptom severity. For example, ‘Sadness’ has to be judged on a scale from 0 = ‘I do not feel sad’ to 3 = ‘I am so sad or unhappy, that I can't stand it’. Scores are summarized and values between 11 and 17 are regarded to be ‘heightened’, and higher scores are valued as ‘clinically relevant’ (30). The Cronbach's α is reported to be 0.74 for healthy subjects and 0.92 for depressed patients (30). The STAI and the BDI were completed to provide the psychologist with more information on trait anxiety and depressive symptomatology. Participants scoring in a clinical range were excluded from the sample.

Immediately after diagnostics, participants underwent an experimental session. They were exposed to a total of 120 pictures representing four different emotional categories – ‘Phobia’, ‘Danger’, ‘Disgust’, and ‘Neutral’ – during a combined recording of the EMG and the electrocardiogram (ECG). Pictures were selected from the International Affective Picture System (IAPS; (31)) and a picture set belonging to the authors 12, 13. The phobia-related stimuli depicted scenes of dental treatment. Disgust-relevant pictures represented different domains, such as ‘repulsive animals’ or ‘poor hygiene’. Danger-related pictures showed predators (e.g. shark or lion) or attacks by humans (e.g. with knives or pistols), whereas neutral pictures consisted of household articles or geometric figures. The pictures were shown in random order for 6,000 ms each. Interstimulus intervals varied randomly between 8,000 and 12,000 ms. Immediately after the picture-viewing experiment, participants rated their impression of the pictures (on a whole for one picture category, but separately for each category) using the Self-Assessment Manikin (SAM; (32)). We employed three scales: ‘arousal’, ‘fear’, and ‘disgust’ (e.g. ‘Please rate how aroused you felt during the presentation of these pictures’). Participants had to rate their ‘arousal’ on a visual scale which consisted of manikins showing increasing arousal from ‘1’ indicating no arousal to ‘9’ indicating massive arousal. The dimensions ‘disgust’ and ‘fear’ were rated on two, nine-point Likert scales ranging from 1 to 9, with ‘1’ indicating that the subject felt no anxiety or disgust and ‘9’ indicating that the subject felt very anxious or disgusted. This was used as a ‘manipulation check’ to ensure that phobics felt more aroused by dental treatment scenes compared with controls and that they really experienced higher levels of fear and disgust. The SAM is a very quick and non-verbal method for assessing emotional experience. According to the authors (32) the SAM correlates highly with ratings obtained using a verbal, more lengthy, semantic differential scale.

### Data recording and analysis

Psychophysiological data were recorded using a Brain Amp 32 system (Brain Vision, Munich, Germany). Data were sampled at 2,500 Hz and the passband was set to 0.016–70 Hz.

For the EMG, electrodes were placed on the levator labii muscle region, according to previous guidelines (33). All impedances of the EMG electrodes were below 10 kΩ. Unipolar EMG channels were transformed to bipolar montages. Data were visually inspected for artifacts, and in preparation for statistical analysis were bandpass filtered (30 - 500 Hz, 24 dB/octave), rectified, and low-pass filtered (8 Hz, 24 dB/octave). Smoothed EMG segments from individual stimuli were baseline corrected by a 1,000 ms prestimulus baseline. Activity in the 2,000- to 6,000-ms time period following picture onset was averaged.

The ECG was recorded according to Einthoven II with two Ag/AgCl electrodes. All impedances of the ECG electrodes were below 30 kΩ. The signal was amplified (high pass: 0.5 Hz) and an automatic peak trigger detector was used to identify R-waves. Data were baseline-corrected with a 4,000-ms period before picture onset and activity between 2,000 and 6,000 ms after picture onset was averaged separately for categories.

For statistical data analyses, Version 20 of IBM SPSS Statistics (International Business Machines, Armonk, NY, USA) was used. Questionnaire data were submitted separately to two-way anovas with the factors group (phobics and controls) and sex (men and women). There were slight baseline differences between the phobic group and the control group concerning their physiological reactions to neutral pictures. Therefore, differences between responses to emotional and neutral pictures were analyzed (e.g. Phobia minus Neutral). Affective ratings (experienced arousal, fear, and disgust), EMG amplitudes of the levator labii, and heart rate were submitted separately to three-way anovas with the factors group (phobic and control), sex (men and women), and category (Phobia minus Neutral, Disgust minus Neutral, and Danger minus Neutral). For the anovas, Greenhouse–Geisser correction was applied if appropriate. For all anovas we reported partial *η*^2^ as effect sizes. To clarify significant interactions, further analyses were conducted using *t*-tests.

## Results

### Questionnaire data and affective ratings

The two-way anova revealed a significant main effect of *group* for DAS scores (*F*_1,68_ = 471.3, *P* < 0.001, *η*^2^ = 0.874). Phobic subjects had higher scores than controls (see Table 1). Moreover, there were significant main effects of *group* for STAI scores (*F*_1,68_ = 6.5, *P* = 0.013, *η*^2^ = 0.088) and BDI scores (*F*_1,68_ = 11.7, *P* < 0.001, *η*^2^ = 0.147) with phobic subjects showing higher scores than controls. However, in both questionnaires all groups scored in a non-clinical range, which means that they did not show heightened trait anxiety or symptoms of depression. Consequently, we did not compute further analyses based on these questionnaires. There was a significant main effect of *sex* for the QADS, with female subjects displaying higher scores than male subjects (*F*_1,68_ = 20.0, *P* < 0.001, *η*^2^ = 0.227). No other main effects or interactions reached statistical significance (all *P* > 0.083).

**Table 1:** Participants’ scores on the Dental Anxiety Scale (DAS), the Questionnaire for the Assessment of Disgust Sensitivity (QADS), the State-Trait Anxiety Inventory (STAI), and the Beck Depression Inventory (BDI), as well as affective picture ratings (responses to Neutral pictures have been subtracted)

	Group					
	Phobic subjects			Controls		
Variable	Both sexes	Men	Women	Both sexes	Men	Women
Questionnaires						
DAS	16.8 ± 2.0	16.3 ± 2.1	17.3 ± 2.0	7.2 ± 1.7	7.5 ± 1.6	6.9 ± 1.8
QADS	1.9 ± 0.7	1.5 ± 0.6	2.3 ± 0.5	1.7 ± 0.6	1.5 ± 0.6	1.9 ± 0.5
BDI	5.9 ± 5.2	6.4 ± 6.4	5.3 ± 3.7	2.5 ± 2.8	2.9 ± 2.9	2.1 ± 2.6
STAI	36.6 ± 10.6	36.8 ± 11.4	36.4 ± 10.0	31.3 ± 6.5	31.4 ± 6.6	31.1 ± 6.6
Picture ratings						
Phobia minus Neutral						
Arousal	4.9 ± 1.6	4.6 ± 1.5	5.3 ± 1.6	0.9 ± 1.5	0.9 ± 1.3	0.9 ± 1.7
Fear	5.1 ± 2.0	4.7 ± 1.9	5.5 ± 2.0	0.5 ± 0.7	0.5 ± 0.6	0.6 ± 0.8
Disgust	2.6 ± 2.4	2.0 ± 2.3	3.1 ± 2.4	0.5 ± 0.6	0.6 ± 0.6	0.5 ± 0.6
Disgust minus Neutral						
Arousal	3.4 ± 2.1	2.9 ± 2.1	3.9 ± 2.0	3.4 ± 2.4	2.7 ± 1.7	4.2 ± 2.8
Fear	1.6 ± 1.7	1.3 ± 1.8	1.9 ± 1.7	1.1 ± 1.3	0.8 ± 1.1	1.3 ± 1.5
Disgust	5.6 ± 2.4	4.7 ± 2.7	6.6 ± 1.5	5.2 ± 2.0	4.8 ± 2.0	5.6 ± 1.9
Danger minus Neutral						
Arousal	2.4 ± 1.7	2.0 ± 1.6	2.8 ± 1.7	2.9 ± 2.2	3.6 ± 1.8	2.2 ± 2.5
Fear	2.5 ± 1.9	1.8 ± 1.5	3.2 ± 2.1	2.3 ± 1.8	2.6 ± 1.7	2.1 ± 1.9
Disgust	0.8 ± 1.3	0.8 ± 1.4	0.8 ± 1.2	0.6 ± 0.9	0.6 ± 0.8	0.7 ± 0.9

Values are given as mean ± SD.

The three-way anova for ratings of perceived arousal revealed significant interactions for *group x sex x category* (*F*_2,136_ = 3.8, *P* = 0.024, *η*^2^ = 0.053), *group x category* (*F*_2,136_ = 55.6, *P* < 0.001, *η*^2^ = 0.450), and *sex x category* (*F*_2,136_ = 4.9, *P* = 0.009, *η*^2^ = 0.067), as well as significant main effects for *group* (*F*_1,68_ = 10.8, *P* = 0.002, *η*^2^ = 0.137) and *category* (*F*_2,136_ = 5.4, *P* = 0.006, *η*^2^ = 0.073). For fear there was a significant interaction for *group x category* (*F*_2,136_ = 58.7, *P* < 0.001, *η*^2^ = 0.463), as well as significant main effects for *group* (*F*_1,68_ = 41.1, *P* < 0.001, *η*^2^ = 0.378) and *category* (*F*_2,136_ = 22.9, *P* < 0.001, *η*^2^ = 0.252). For disgust there were significant interactions for *group x category* (*F*_1.6,111.2_ = 9.2, *P* = 0.001, *η*^2^ = 0.119) and *sex x category* (*F*_1.6,111.2_ = 3.8, *P* = 0.033, *η*^2^ = 0.053), as well as significant main effects for *group* (*F*_1,68_ = 9.0, *P* = 0.004, *η*^2^ = 0.117), *sex* (*F*_1,68_ = 4.8, *P* = 0.033, *η*^2^ = 0.065), and *category* (*F*_1.6,111.2_ = 227.3, *P* < 0.001, *η*^2^ = 0.770). No other main effects or interactions reached statistical significance (all *P* > 0.094).

Furthermore, between-group *t*-tests for each category showed significant differences between phobic subjects and controls for Phobia minus Neutral only. Phobic subjects reported higher arousal (*t*_70_ = 11.7, *P* < 0.001), fear (*t*_44.5_ = 14.1, *P* < 0.001), and disgust (*t*_39.6_ = 5.2, *P* < 0.001) than controls when confronted with dental treatment scenes (see Table 1). No group differences were found for the other picture categories (all *P* > 0.067).

Between-group *t*-tests for each category showed sex effects within phobic subjects: women and men differed significantly in their disgust reports to Disgust minus Neutral (*t*_26.6_ = 2.6, *P* = 0.015) and fear reports to Danger minus Neutral (*t*_34_ = 2.2, *P* = 0.034). In both cases, women showed higher scores than men. All other tests were non-significant (all *P* > 0.139). There were no effects of sex within controls (all *P* > 0.070).

### EMG of the *musculus levator labii*

The three-way anova revealed significant interactions for *group x sex x category* (*F*_2,136_ = 3.1; *P* = 0.045, *η*^2^ = 0.043), *group x sex* (*F*_1,68_ = 4.1; *P* = 0.047, *η*^2^ = 0.57), and *group x category* (*F*_2,136_ = 3.8; *P* = 0.025, *η*^2^ = 0.053), as well as a significant main effect of *group* (*F*_1,68_ = 8.8; *P* = 0.004, *η*^2^ = 0.115). No other main effects or interactions reached statistical significance (all *P* > 0.097).

**Fig 1:**
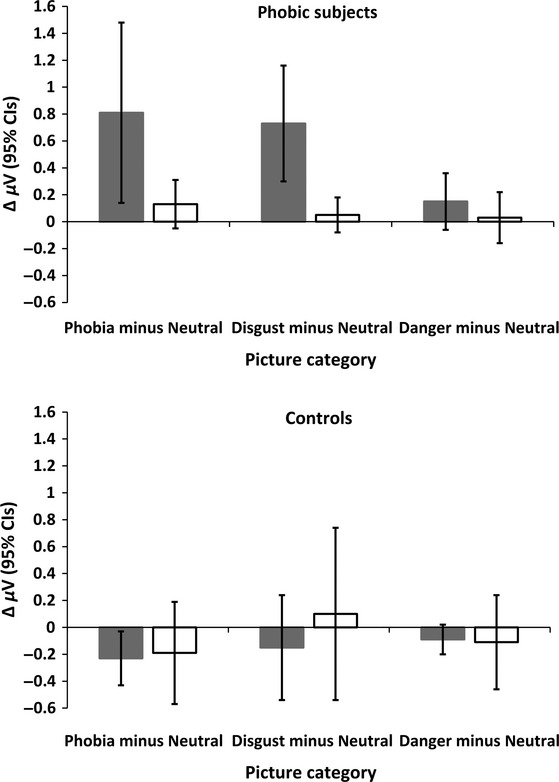
Amplitudes of the *musculus levator labii* showing responses to the three emotional categories minus responses to affectively neutral pictures (Phobia minus Neutral, Disgust minus Neutral, and Danger minus Neutral) for phobic subjects and controls. Grey bars, women; white bars, men.

Further between-group *t*-tests for each difference showed significant effects for Phobia minus Neutral pictures only (*t*_70_ = 3.4, *P* = 0.001). Phobic subjects displayed higher activation than controls when confronted with dental treatment scenes (Fig. 1). No effects were found for the other differences (all *P* > 0.056).

A one-way anova within women revealed a significant *group x category* interaction (*F*_2,68_ = 4.7; *P* = 0.012, *η*^2^ = 0.122) and a significant main effect for *group* (*F*_1,34_ = 12.4; *P* = 0.001, *η*^2^ = 0.267), whereas there were no significant effects in men (all *P* > 0.253).

Within phobic subjects, *t*-tests between both sexes showed greater activation differences in women compared with men in response to Phobia minus Neutral (*t*_19.4_ = 2.1, *P* = 0.048) and Disgust minus Neutral (*t*_20.0_ = 3.1, *P* = 0.005). There were no sex differences within controls (all *P* > 0.472).

### ECG data

The three-way anova revealed a significant interaction for *group x category* (*F*_1.7,115.2_ = 11.6; *P* < 0.001, *η*^2^ = 0.145) and significant main effects of *group* (*F*_1,68_ = 18.3; *P* < 0.001, *η*^2^ = 0.212) and category (*F*_1.7,115.2_ = 16.5; *P* < 0.001, *η*^2^ = 0.195). No other main effects or interactions reached statistical significance (all *P* > 0.258). Note that there was no interaction with *sex* or main effect of *sex*.

**Fig 2:**
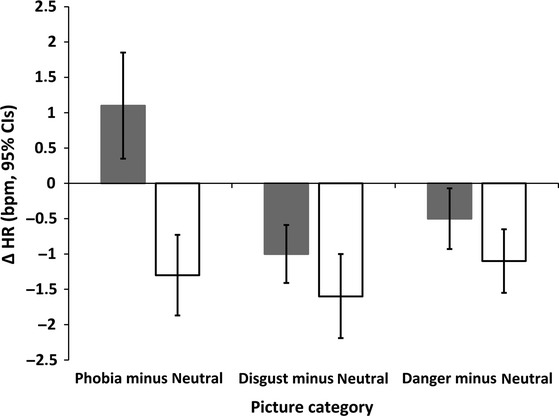
Heart rate of phobic subjects and controls showing responses to the three emotional categories minus responses to affectively neutral pictures (Phobia minus Neutral, Disgust minus Neutral, and Danger minus Neutral). Grey bars, phobic subjects; white bars, controls. bpm, beats per minute.

Further *t*-tests between phobic subjects and controls for each category showed significant group differences for Phobia minus Neutral pictures only (*t*_70_ = 5.3, *P* < 0.001). Phobic subjects displayed greater heart-rate acceleration than controls (who displayed heart-rate deceleration) when confronted with dental treatment scenes (Fig. 2). No group differences were found for the three other picture categories (all *P* > 0.059).

## Discussion

This study investigated sex differences in changes of heart rate and facial EMG activity of the *musculus levator labii* in patients with dentophobia. Whereas men and women did not differ in their self-ratings of arousal, fear, and disgust or in heart-rate acceleration when confronted with dental treatment scenes, there were sex differences in the facial EMG activity. Only women showed a rise in *musculus levator labii* activity.

It is a widely known stereotype, which seems to exist in most cultures, that women are more ‘emotional’ than men (34). There is one published investigation on how frequently participants believed that women and men performed specific nonverbal behavior (35). Women were expected to express more nonverbal emotional behavior than men, and to be more skilled at sending and receiving nonverbal messages. This is also true when people are asked to judge their ideologies of how people should deal with emotions in relationships. In an investigation, women favored a direct expression of emotion, whereas men appreciated emotional management (36). There is, indeed, a substantial body of evidence suggesting that women are more emotionally expressive than men [for a review, see (37)]. However, whether women are only more emotionally expressive, or if they really experience stronger emotions, remains unresolved. Studies that found no differences in self-reports of emotion between men and women strongly speak against the theory that women experience stronger emotions. For example, one study (38) presented participants with emotion-evoking film clips and men and women did not differ in reporting how emotional they felt. However, psychophysiological reactions sometimes do not mirror verbal reports of emotional experience. For example, in the aforementioned study, inconsistencies between verbal reports of emotional experience and facial expression of the same emotion have been reported, as women more frequently showed facial expressions of emotions than did men. A study found a similar result as women, relative to men, showed higher facial EMG activity of the *musculus corrugator supercilii* during presentation of unpleasant pictures (39).

It might therefore be possible that psychophysiological indicators are more accurate measures of emotional experience than are verbal self-reports. This has already been documented by Schienle *et al*. (12, 13), who observed sex-specific brain activation in dentophobic men and women during symptom provocation, whereas verbal self-reports of emotions did not differ between the sexes. A study employing the electroencephalogram (EEG) reported enhanced late frontal positivity in dentophobic men relative to dentophobic women in a time window between 300 and 1,500 ms, which was interpreted to reflect a sex-dependent recruitment of frontal attentional networks and an advantage for men concerning emotion regulation abilities (12). A subsequent investigation employing functional magnetic resonance imaging (fMRI) showed similar results (13). Dentophobic men displayed enhanced activity of the dorsolateral prefrontal cortex, which was negatively correlated with arousal experienced, possibly reflecting active engagement in cognitive control strategies. In contrast, dentophobic women showed greater activation of the caudate nucleus and were characterized by a greater caudate volume relative to phobic men, which, moreover, was positively correlated with arousal experienced during exposure and symptom severity. This result might mirror a greater tendency to rehearse disorder-relevant stimulus-response associations in women. Dentophobic subjects are characterized by a high frequency of catastrophic cognitions (e.g. ‘The drill will be painful’ or ‘I have no control’). These cognitions are part of a vicious cycle leading to the maintenance of the disorder (27). Possibly, men are more able to inhibit negative automatic cognitions during dental treatment than women. This, however, remains to be tested.

Within the current study, we were unable to find sex differences in emotional experience according to self-ratings. However, although dentophobic men perceived dental treatment scenes as equally disgust-inducing as did dentophobic women, they displayed significantly lower disgust-related facial EMG activity. It might be possible that men more successfully inhibit behavioral reactions during confrontation. This agrees with the aforementioned EEG and fMRI results and indicates that men more successfully regulate their reactions during symptom provocation. One might also argue that in women the dentophobic reaction goes beyond fear and involves feelings of disgust. It remains to be tested if dentophobic women display more disgust-related negative automatic cognitions (e.g. the fear of gagging during dental treatment). However, additional therapeutic strategies targeting feelings of disorder-specific disgust might be of great value for dentophobic women.

Additionally, it has to be noted that the heart-rate acceleration during symptom provocation, observed herein, does not match the psychophysiological reaction pattern typically observed in specific phobia of the BII subtype. This has already been reported for dentophobic women (8) and also seems to be the case for dentophobic men. Therefore, the classification of dentophobia within the BII subtype of specific phobia, according to the DSM-IV-TR (24), should be reconsidered.
